# The South African National HIV Pregnancy Cohort: evaluating continuity of care among women living with HIV

**DOI:** 10.1186/s12889-020-09679-1

**Published:** 2020-11-05

**Authors:** Kate Clouse, Babatyi Malope-Kgokong, Jacob Bor, Cornelius Nattey, Maanda Mudau, Mhairi Maskew

**Affiliations:** 1 Vanderbilt University School of Nursing, Nashville, TN USA; 2 Vanderbilt Institute for Global Health, Vanderbilt University Medical Center, Nashville, TN USA; 3 Health Economics and Epidemiology Research Office (HE2RO), Department of Internal Medicine, School of Clinical Medicine, Faculty of Health Sciences, University of the Witwatersrand, Johannesburg, South Africa; 4 Academic Affairs and Research, National Health Laboratory Service, Johannesburg, South Africa; 5 Department of Global Health, Boston University School of Public Health, Boston, MA USA; 6 Department of Epidemiology, Boston University School of Public Health, Boston, MA USA

**Keywords:** HIV/AIDS, Pregnancy, Postpartum, Women living with HIV/AIDS, South Africa, Big data, National cohort

## Abstract

**Background:**

South Africa is home to more people living with HIV than any other country, including nearly one in three pregnant women attending antenatal care. Access to antiretroviral therapy (ART) has increased substantially since the start of the national ART program in 2004, with > 95% ART coverage during pregnancy and delivery, and vertical transmission of HIV greatly reduced. However, women who initiate ART during pregnancy are at heightened risk of dropping out of care, particularly after delivery, leading to the potential for viral transmission, morbidity and mortality. It is difficult to evaluate the success of policies of expanded access to ART care, and assess continuity of care, due to the lack of a national longitudinal HIV care database. Also, patient movement between unlinked facilities. For the first time on a national level, we propose to utilize routinely-collected laboratory data to develop and validate a cohort of pregnant women living with HIV in South Africa in a way that is uniquely robust to facility transfer.

**Methods:**

Using laboratory test data matched to facility type, we will identify entry to antenatal care to build the cohort, then describe key treatment milestones, including 1) engagement in antenatal care, 2) initiation of ART, 3) HIV viremia, and 4) continuity of HIV care in the postpartum period. Second, we will measure the effect of system-wide factors impacting continuity of care among pregnant women. We will assess policies of expanded treatment access on continuity of care using regression-discontinuity analyses. We then will assess mobility and its effect on continuity of care during and after pregnancy. Third, we will identify individual-level risk factors for loss from HIV care in order to develop targeted interventions to improve engagement in HIV care.

**Discussion:**

This work will create the world’s largest national cohort of pregnant women living with HIV. This novel cohort will be a powerful tool available to policymakers, clinicians and researchers for improving our understanding of engagement in care among pregnant women in South Africa and assessing the performance of the South African national ART program in caring for pregnant women living with HIV.

**Trial registration:**

N/A (not a clinical trial).

## Background

South Africa is home to more people living with HIV than any other country in the world [1] – over 7 million people [2] – and the national ART program, which started in 2004, is the world’s largest [3]. Since 2016, South Africa has implemented a “treat all” policy, providing ART to all HIV-positive people, regardless of CD4 cell count [3]. This rapid-scale up has undoubtedly had a positive effect on morbidity and mortality due to HIV, as well as HIV transmission: there was an estimated life expectancy gain of 11.3 years between 2003 and 2011 due to ART [4] and a 77% decrease in HIV transmission in stable serodiscordant couples [5]. However, deaths from AIDS-related causes remain high – over 70,000 in 2018 [6] – and the public healthcare system has demonstrated challenges with meeting the sustained need for initiating and retaining patients on lifelong ART. Poor long-term engagement in HIV care within the public sector in South Africa is a known problem [7, 8], and one that threatens to minimize the gains made by these ambitious national policies.

Black African women of reproductive age (ages 20–34) are the population most heavily affected by the South African HIV epidemic, with an estimated 31.6% prevalence [9]. Through policies of expanded ART, including Option B+ (2015) and “treat all,” South Africa has nearly eliminated vertical transmission of HIV [10]. However, recent work has shown that women who initiate ART during pregnancy are at very high risk of loss to follow-up [11–15], with re-engagement in routine HIV care after delivery particularly difficult [16, 17]. HIV and tuberculosis (usually exacerbated by HIV) are the top two causes of death among women ages 15–44 in South Africa [18], demonstrating that a focus on sustained engagement in care must continue after pregnancy ends.

South Africa slowly is moving towards adopting national health insurance, and with it, national data systems and a unique identifier. However, from the dawn of the AIDS crisis to now, South Africa has had no centralized data collection system, resulting in a fragmented health care system is not adapted for a mobile population. Traditionally, records have been paper-based, and recently ART programs have moved to retrospectively capturing data in usually-proprietary database programs. This disparate nature of data capture and storage impacts estimates of retention in HIV care, as patients may move between facilities as “silent transfers,” but their data will not [19–21]. Such misclassification is an impediment to evaluating the effectiveness of ART programs, and also prohibits real-time sharing of data between clinicians at different clinics.

South Africa’s population is highly mobile, with a long history of circular movement of people between rural and urban areas [22, 23]. Our work has shown that urban, postpartum women in South Africa are a highly mobile group, often experiencing long-distance travel to visit family member in rural areas and seek help with the baby [19, 24]. The impact of mobility on engagement in HIV care also has been observed in Kenya [25, 26], Lesotho [27], Zambia [28], and the US [29]. In South Africa, there is a broken continuum of care between antenatal care at a primary care clinic, labor and delivery at a hospital or designated obstetric facility, and a return to routine HIV care at the primary care clinic. Improved pregnancy-related data systems are necessary to effectively characterize the HIV continuum of care among pregnant women and to evaluate the national ART programs effectiveness of treating pregnant and postpartum women.

### Objectives

#### Aim 1: develop and validate a novel national cohort of pregnant women accessing HIV care

Utilizing data on specific laboratory test and facility type available in the NHLS National HIV cohort, we will identify results of blood tests taken during antenatal services. We then will use external non-laboratory cohorts with known antenatal care data as gold standard datasets to validate imputed pregnancy-related variables created in the NHLS National HIV cohort. In this way, we will build a national-level pregnancy cohort and describe key treatment milestones in the care of pregnant women living with HIV in South Africa: 1) engagement in antenatal care, 2) initiation of ART, 3) HIV viremia and 4) continuity of HIV care in the postpartum period, a period of high LTFU.

#### Aim 2: measure the effect of system-wide factors impacting continuity of care among pregnant women

Timing of presentation with HIV in relation to pregnancy, as well as mobility in the peripartum period, are critical yet understudied phenomena in the pregnancy treatment cascade. While most cohorts in South Africa first observe HIV patients at initiation of ART, our pregnancy cohort will observe patients from first CD4 count regardless of where they seek antenatal care and, most importantly, where they continue in care postpartum.

2a. Policies of expanded treatment access. We will estimate the effect of expanded treatment access on engagement and continuity in HIV care. Describing national trends, we will assess how these impacts have been sustained or improved. We hypothesize that engagement in HIV care during pregnancy continues to improve in the era of expanded access but long-term retention of women on ART after pregnancy does not.

2b. Assess mobility and its effect on continuity of care both during and after pregnancy. We will describe mobility as it relates to ART treatment and measure the impact on continuity of care. We hypothesize mobility among pregnant women is frequent and predicts subsequent disengagement from HIV care.

#### Aim 3: identify individual-level risk factors for loss from HIV care

Designing strategies to address losses from care requires knowledge of who is most at risk of loss from care. Utilizing machine learning and advanced statistical techniques, we will harness the national scope of our HIV pregnancy cohort with clinical data from external HIV cohorts to build a predictive model identifying pregnant women most at risk of disengaging from HIV care. We hypothesize that modifiable factors will be identified to inform strategies to reduce loss from care.

### Study design

Longitudinal cohort study

## Methods

### Data sources

This proposal will leverage two key observational data sources to achieve the project aims:

#### NHLS national HIV cohort

As the central laboratory system for South Africa’s public sector, the NHLS has provided laboratory and pathology services to an estimated 80% of the national population since 2004 (with the exception of KwaZulu-Natal, which joined in 2009). The data stored in the NHLS Corporate Data Warehouse (CDW) are captured electronically from each individual laboratory requisition form which includes demographic (name, surname, sex and date of birth), geographic and facility-specific variables (province, sub-district, facility name, ward name and type). The CDW is the national laboratory repository storing HIV monitoring tests, including CD4 count, viral load and other lab results. A key advantage of these data is that the electronic lab records are generated through routine patient care and do not depend on an additional step of data capture and entry at the clinic level, which comes with attendant increased risks of error. The CDW includes identifying information about the patients (e.g., name, sex, date of birth, etc.), information about the clinic where the test was performed (e.g., clinic ID) and information on the results of the test (e.g., date of testing, test result).

As the participant records captured in the CDW lack a unique patient identifier, a linkage algorithm was developed to identify unique patients (and their associated laboratory results) through the demographic data available using probabilistic and network-based linkage methodology, creating the NHLS National HIV Cohort. To date, this linkage is only done internally within the NHLS database (i.e., we linked CD4 tests to VL tests from the same individuals, regardless of the date the sample was taken) and, as such the NHLS National HIV Cohort has not been linked externally to other databases. In this project, we propose to create a National HIV Pregnancy Cohort that is nested within the larger NHLS National HIV Cohort. To do that, we will link rapid plasma reagin (RPR) syphilis and rhesus factor tests to the NHLS National Cohort to identify a sub-set of pregnant women nested within the larger NHLS National HIV Cohort. The sample size for this analysis will be the entire NHLS National HIV cohort that currently includes 56,000,000 test results associated with nearly 8.5 million unique IDs with female sex. This includes 19.5 million CD4 counts and 13.5 million VL from the 6.3 million women aged 15–45. Permission to analyze these data has been obtained.

#### Validation cohorts

We will identify non-laboratory clinical cohort datasets that contain information on HIV testing (where available), antenatal booking date and care visits, antiretroviral treatment record as well as laboratory data from the NHLS relevant to the patient cohort including test type, date and result.

### Eligibility criteria

Data will be included in the cohort and analysis if:

- Data were collected as part of the public sector HIV treatment program in South Africa
- Lab test was performed on or after April 1st, 2004, when the public sector treatment program began scale-up
- Lab test conducted on female patients (regardless of whether that particular test result was coded as female)

Study exclusion criteria will be:

- Lab test was performed before April 1st, 2004

We will not restrict to any minimum or maximum age of research subjects. Though we do not expect to observe pregnancy among female infants and toddlers, we plan to include all data from female patients so that entry to HIV care and ART treatment prior to pregnancy (including during childhood and adolescence) may be observed and accounted for during analyses.

### Procedure for informed consent

As the study will utilize only routinely collected laboratory data, no individual patient consent with be sought.

### Data collection and analysis plan

#### Aim 1: develop and validate a novel national cohort of pregnant women accessing HIV care

We propose to utilize the NHLS National HIV Cohort to create and validate a national, longitudinal, patient-level cohort of pregnant women accessing HIV care in South Africa dating to the beginning of the national ART program in 2004, as follows:

- **Creation of patient-level longitudinal cohort using pregnancy indicators** – The development of the NHLS National HIV Cohort using only laboratory data from the NHLS resulted in an initial database linking all lab tests to individuals based upon an exact link to surname, first initial of first name and date of birth. This patient matching method was validated by applying the same algorithm to a known cohort of over 36,487 unique individuals with associated clinical and laboratory data – the Themba Lethu Clinical Cohort in Johannesburg [30]. This validation exercise resulted in over-linking (i.e., inappropriately matched results) only 41 individuals. For this study, we plan to utilize indicators of pregnancy status within the existing NHLS National HIV Cohort in order to create a National HIV Pregnancy Cohort of women living with HIV. We will utilize the entire dataset of laboratory tests associated with a female sex and code each sample as either associated with a pregnant or non-pregnant woman. We will identify the subset of pregnant women nested within the NHLS National HIV Cohort through the following indicators:

1. ***Identification of blood samples arising from antenatal facilities***. While the NHLS National HIV Cohort does not contain clinical data from patient visits for antenatal care, specific laboratory tests are performed routinely for pregnant women at entry to antenatal care including:
  - *Hemoglobin (Hb):* indicated at the first antenatal visit to check for maternal anemia.
  - *RPR Syphilis*: Maternal infection with syphilis represents a significant risk to the fetus, so early detection and treatment can help prevent pregnancy loss due to syphilis infection.
  - *Rhesus factor identification:* Rhesus status is determined at first antenatal visit to identify Rh negative women who may require management for Rh antibody production against the red blood cells of a Rh-positive fetus.
  - *CD4 count for women either known or newly diagnosed with HIV*: According to national guidelines, all pregnant women diagnosed with HIV should be initiated onto lifelong ART at entry to antenatal care. Baseline CD4 counts are taken at initiation of ART.
  While none of these tests on their own are 100% specific for pregnancy (though the specificity of Rhesus testing is close to 100%), combinations of these tests performed on the same day is very strong evidence supporting the assumption of the test date as the first antenatal visit.
2. ***Facility identifiers****.* Data identifying the facility from which each sample originated are captured with each requisition form submitted with blood samples to the NHLS. In addition to the facility name, variables detailing the ward name and ward description are also recorded for each associated blood sample. This data creates two valuable data points for the National HIV Pregnancy Cohort:
  - *Identification of pregnancy care*: Blood samples submitted with a requisition form indicating the ward from which the sample originated will be analyzed and coded to determine if the text descriptor identifies the sample as originating from an antenatal source. Blood samples arising from antenatal facilities will be assumed to be tests taken as part of routine antenatal care for pregnant women.
  - *Mobility indicator*: Patient movement from one facility to another is difficult to track with a traditional facility-based cohort. Using the facility identifier associated with each blood sample taken as described above, our National HIV Pregnancy Cohort will be able to, for the first time, directly and accurately observe patient movement across facilities at a national level regardless of the distance of transfer. The geographic information available in the laboratory data enables characterization of movement at the level of the facility, district and province.

- **Data validation** – Once we have determined pregnancy status for each blood sample as described, we will validate the ascertainment of pregnancy status by linking a subset of the National HIV Pregnancy Cohort to the validation patient cohorts on patient identifiers constructed in the matching process. By linking the matched NHLS cohort with the validation datasets at the individual level, we can measure the precise levels of under- and over-ascertainment of pregnancy. We also can obtain the optimal combination strategy of pregnancy indicators that allows for creation of a patient-level pregnancy cohort from the NHLS that closely resembles the validation cohort data.
- **Variable definitions** – Once we have successfully ascertained patient-level pregnancy status, we will use the associated data features to define the following key treatment milestones and other variables:
  - ○ *Entry to HIV care*: Date of first observed CD4 count in the NHLS data.
  - ○ *ART initiation*: Although dates of ART initiation are not directly recorded in the labs data, we can infer date of initiation based on routine monitoring blood tests taken immediately prior to, or at ART initiation according to national guidelines. We previously validated this methodology against cohorts with known ART start dates [31] and achieved sensitivity and positive predictive values of > 90%.
  - ○ *Entry to antenatal care*: Date of first observed Rh and RPR test in combination with a CD4 count/Hb.
  - ○ *Continuity of HIV care:* As dates of death are not available, the primary outcome of continuity of HIV care is defined as death and loss to follow-up. We will define a patient as lost from care on the first date that they are ≥3 months late for a scheduled lab test, as per national guidelines. So, for example, if a patient has a lab test scheduled (under guidelines) to occur at 12 months post-ART initiation and they reach month 15 with no viral load, they would be considered LTFU.
  - ○ *Re-engagement in HIV care:* As we expect that pregnant women may have repeated episodes of loss followed by re-engagement in care, we will also use a second definition of overall loss from care as the last date a patient is observed as lost in the dataset. This second definition allows patients to re-enter the sample by returning to care.
  - ○ *Mobility:* We will define a patient as transferred to another clinic on the date when they have a first observed blood test at a new treatment clinic.
  Viral loads and CD4 counts are routinely collected for all patients in the national ART program according to national guidelines. In the matched cohort, we will have pre-ART CD4 counts, CD4 counts and viral loads at initiation (depending on the year) and six-monthly or annual CD4 counts and viral loads thereafter (depending on the year). Because we observe patients regardless of self-transfer, we will be able to follow patients’ viral loads and CD4 counts for longer than most other studies, and without this potentially important source of bias.

##### Aim 1 outcomes and analysis

Table 1 summarizes the anticipated outcomes of Aim 1. We will commence analysis with a descriptive summary of the demographic and clinical characteristics of women included in the sample. We will then conduct a crude analysis reporting simple proportions with 95% confidence intervals of patients achieving each outcome. Next we will compare the proportions of women achieving important ART treatment cascade milestones (initiation of ART, HIV viremia and continuity of care) by pregnancy status: 1) pregnant at entry to HIV care; 2) observed pregnancy after entry to HIV care; and 3) no observed pregnancy. [Though women without an observed pregnancy will not be included in the National HIV Pregnancy Cohort, we will use the group of non-pregnant women within the NHLS national HIV cohort as a comparison group for the Aim 1 analysis.] The analysis will begin with a simple comparison of the three groups with respect to baseline predictors of outcomes to look for any imbalances. These potential confounders include demographic (e.g., age) and clinical variables (baseline CD4 and hemoglobin counts), as well as geographic and facility-level factors (urban vs rural setting, facility type among others). We will then conduct a crude analysis comparing the proportion of patients achieving each dichotomous outcome by group and using a log-linear regression model, we will estimate crude risk ratios and crude risk differences and their corresponding 95% confidence intervals. Should any important imbalances be observed, we will proceed with an adjusted model.

**Table 1:** Aim 1 outcomes

Outcomes	Specific indicators	Study Aim
Engagement in antenatal care (ANC)	Number of women living with HIV (WLWH) entering antenatal care (ANC) each year since 2004	Aim 1
	Trends in age of WHWH first accessing HIV care during ANC	Aim 1
	Number of subsequent pregnancies per WLWH	Aim 1
Initiation of antiretroviral therapy (ART)	Proportion of WLWH receiving pre-ART blood tests during ANC	Aim 1
HIV viremia	Proportion of WLWH with major (> 1000 copies/mL) and minor (50–1000 copies/mL) viremia during ANC	Aim 1
	Proportion of women with major (> 1000 copies/mL) and minor (50–1000 copies/mL) viremia within first 2 years postpartum	Aim 1
	Association between viremic episodes and timing of ART initiation	Aim 1
Continuity of HIV care	Proportion of WLWH continuously in care (≤12 months between visits) over study period	Aim 1
	Proportion of WLWH only seen at ANC and then lost after first pregnancy (> 12 months since last visit)	Aim 1
	Proportion of WLWH who only engage in HIV care during pregnancy episodes (first and subsequent)	Aim 1
	Proportion of WLWH who re-engage in care after LTFU (> 12 months)	Aim 1
	Geographic distribution of continuity of care	Aim 1

#### Aim 2: measure the effect of system-wide factors impacting continuity of care among pregnant women

Using the National HIV Pregnancy Cohort longitudinally, including 15 years of South Africa’s public-sector ART rollout, we will determine the short- and long-term effects of the implementation of each progressive step of expanded treatment access, as well as the durability of these effects over time (Aim 2a). In addition, we will be able to report the only national estimates of mobility across geographic regions and facility types as well as measure the impact of mobility on continuity of care among pregnant women (Aim 2b).

##### Aim 2 outcomes and statistical analysis

Aim 2 outcomes are displayed in Table 2. We will estimate system-wide continuity of care in both the pregnancy and postpartum period and describe where important losses occur. Starting on the date of a patient’s entry to HIV care (first CD4 count), we will estimate the proportion and cumulative probabilities of ART initiation, transfer, and loss to follow up and report them with corresponding 95% confidence intervals, as well as with simulation intervals that account for sources of bias. We will also stratify our analyses by district and sub-district to look for differences across geographic areas within South Africa.

**Table 2:** Aim 2 outcomes

Outcomes	Specific indicators	Study Aim
Effect of expanded ART access policies on clinical outcomes	Aim 1 continuity of care and viremia outcomes stratified by policy time period: Single-dose nevirapine (sdNVP, 2002) Option A (2008) CD4+ threshold raised from 200 to 350 for pregnant women (2010) Option B (2013) Option B+ (January 2015) Treat all (universal ART, 2016)	Aim 2a
Mobility	Proportion of women continuously in care at ANC and one clinic (no switching)	Aim 2b
	Median number of facility switches per woman	Aim 2b
	Factors associated with facility switching	Aim 2b
	Spatial distribution of facility switches	Aim 2b
Impact of mobility (mobility as exposure)	Viremic episodes associated facility switching	Aim 2b
	Among WLWH on ART prior to pregnancy, impact of clinic switching on postpartum continuity of care compared to those who did not switch	Aim 2b

Finally, we will use proportional hazard regression to assess determinants of continuity of care and mobility across clinics. Predictors will include region, baseline CD4, calendar year of first CD4 count and other lab values. We will then model CD4 counts and viral loads as time-varying predictors of retention and transfer in our proportional hazards models. In order to better understand movement between clinics within South Africa, we will use spatial analysis to produce maps depicting the direction of movement between clinics with size proportional to the amount of transfer. In addition, we will use causally-robust regression discontinuity designs (RDD) to assess the impact of eligibility expansion (pre-2015 vs. universal access) on continuity in care. We will use two distinct RDD approaches. First, we will compare pregnant women presenting in the pre-2015 period with CD4 counts above/below the CD4 eligibility threshold. This approach will inform whether ART eligibility was a barrier to ART among pregnant women. Second, we will compare pregnant women seeking care before/after the policy change to assess differences in ART uptake and retention including postpartum.

#### Aim 3: identify individual-level risk factors for loss from HIV care

We will conduct predictive analytic modeling using existing HIV programmatic data to rank-order patients based on risk of defaulting from care. We will utilize existing routinely collected program data that is collected to support HIV care and treatment service delivery in four South African provinces – Gauteng, Free State, Mpumalanga and Limpopo Provinces. Data sources for predictive variables will be developed as described in Aim 1 and include a centralized national electronic health database with patient-level data for participating facilities, NHLS laboratory data with patient level treatment outcome data and a merged data set linking the two datasets. Upon linking these data to the NHLS HIV Pregnancy Cohort, this dataset will be de-identified and will be used to create a risk score based on readily-available clinical, laboratory and demographic data at entry to antenatal care. This risk score will allow health care workers to proactively identify women at entry to care who are at high risk for loss and require intervention while still engaged in care.

The study activities for this aim involve the preparation, development and validation of the proof-of-concept predictive model and will be achieved in three phases:

## Data discovery phase

During the discovery phase, we will identify key data sources and engineer new calculated features that could have value in the model based on factors that have previously been shown to affect patient behavior and outcomes.

1. **Modeling Phase:**

- *Model selection* – trialing different machine learning and statistical techniques to see which performs best in the problem space. Various modelling algorithms will be considered and tested during model selection. For binary classification or risk scoring, a number of potential algorithms will be considered including as logistic regression, decision trees, random forest as well as gradient boosted trees.
- *Feature Engineering and Augmentation* – extrapolating features from the existing data as well as augmentation with new data dimensions where possible. Multiple iterations will be performed to fine-tune the process, using the validation set for feedback, and checking for bias, skewed predictions or over-fit. The predictive power of the model will be measured with area under the curve (AUC) tests.
- *Model refinement & testing* – after initial results, we will finalize the most practical set of input features including reviewing the feature importance and relationships between input features. The model can be refined and optimized to work on the fewest number of input features (most parsimonious version) whilst yielding the highest predictive power. This will involve checks of the model’s sensitivity or dependence on certain variables (overfit).

2. **Final Results Phase:**

The evaluation phase considers the performance of and confidence in the model, and the implications of its strengths and weaknesses to the question addressed. This is performed by considering various metrics including accuracy, precision, sensitivity, specificity, prediction balance, depth of file impact, AUC among others.

### Aim 3 outcomes

This process will provide proof-of-concept of the predictive model approach and provide evidence of the expected effect of improved prediction on the LTFU tracing services currently implemented for pregnant women in the national ART program. The completed predictive model will be structured so that it can be integrated into existing service delivery structures and this scalable at a national level with minimal cost implications. This will be achieved by specifically triaging linkage to care officers’ daily list of missed appointments to improve continuity of care.

### Study timeline

Creation of the cohort will commence roughly mid-way through the first year after completing development of the study protocol and obtaining all relevant ethics and site permissions during the first half of Year 1. Development and validation procedures is expected to continue into the first half of Year 2. Analysis will commence upon the completion of the cohort in Year 2 and will extend to the end of the 5-year project period, with manuscript development and other dissemination occurring throughout Years 3–5. Aim 3 activities will take place during Years 3 and 4, with dissemination of results planned for Years 4 and 5.

### Sample size

We will include all lab data from all female patients who meet all the inclusion criteria and do not meet any of the exclusion criteria – no sampling will be done. As this is an observational study, no assignment to treatment groups will occur though we will stratify patients into pregnant and non-pregnant cohorts. No interaction with patients will occur, so no effort will be made to retain patients in the study. Indeed, as retention in care is one of our primary outcomes for this proposal and because most of the data will be already collected, we cannot strive to intervene to keep patients in care beyond those that clinics are normally using at the sites where patients receive their care. The sample size for this analysis will be the entire NHLS National HIV cohort which currently includes 56,000,000 test results associated with nearly 8.5 million unique IDs with female sex. This includes 19.5 million CD4 counts and 13.5 million VL from the 6.3 million women aged 15–45.

### Recruitment

We will use existing data only, and will not directly recruit or enroll participants. The data will be prospectively collected through indirect participation and cohorts will be updated periodically to provide more recent data.

### Plans to promote participant retention and complete follow-up

This analysis involves linkage and analysis of routinely collected observational data only. No direct patient interaction or intervention to promote retention or follow-up will occur during the study. The NHLS HIV Pregnancy Cohort will be created using routinely collected laboratory data and will be linked to external clinical observational cohorts to:

1. validate assumptions applied to variable creation (for example date of entry to antenatal care)
2. develop a predictive model of risk for loss from care.

### Data management

#### Data security

##### Overview

This study will be performed at the Health Economics and Epidemiology Research Office (HE^2^RO) in Johannesburg, the National Health Laboratory Service (NHLS) in Johannesburg, and Vanderbilt University and the Department of Global Health at Boston University in the US. HE^2^RO and NHLS fall under the FWA of the University of the Witwatersrand Human Ethics Review Committee (Medical). No data will be collected or analyzed that are not routinely collected by the NHLS or national program.

##### Use of identifiable data

As described, the NHLS National HIV Cohort was developed by the NHLS and its’ subsidiaries as well as colleagues at Boston University and HE^2^RO using only laboratory data and associated patient identifiers to create a longitudinal patient record. Updates to this cohort to introduce new (more recent) laboratory data are planned to be done periodically during the five-year study period. In addition to this, the activities described under Aim 1 require access to patient identifiers to link the data on RPR and Rhesus to the existing NHLS National cohort. These updates and linkages will be conducted by the team that developed the original cohort within the NHLS under existing data use agreements outlining access to identifiable patient data for linkage purposes. Linkages to data contained in the validation cohorts will be done under the direct supervision of the study PI.

Once data are developed into a cohort, identifiers will be removed from the analysis file and all subjects will be assigned a random study ID. A password-protected linking file allowing the anonymized data to be linked back to the identifiable data will be kept in a separate file at the NHLS in case problems with the analysis dataset are identified that need to be rectified. In addition, all data files that are not anonymized will be password-protected, with access limited to authorized study staff.

### Data access

The study team will have access to identifiable data during the building of the cohort and during portions of the analysis. There is always the possibility of a breach of confidentiality of study materials when conducting research. While this is acknowledged, there is very low likelihood because of the precautions that will be taken to protect confidentiality. Study staff will be trained on the expectations that they are not to disclose any information collected in the study to anyone outside the study team. All identifying information associated with the study participants will be maintained in complex password-protected computers that themselves are kept in locked rooms or cabinets.

### Oversight and monitoring

This study uses existing data only to develop a national cohort of pregnant women living with HIV in South Africa. We will not prospectively enroll participants and there will be no interaction with individual participants. The only data used in the study that could be used to monitor patient safety will be routinely collected lab data which is generated by the national program, not by the study. The data will be prospectively collected through indirect participation and cohorts will be updated periodically during the study period to provide more recent data. The study team will have no interaction with patients and therefore we will have no role in monitoring patient safety. In addition, since the study requires the use of data that is already collected, no data safety and monitoring board will be employed.

## Protection of human subjects

### Ethics approval and consent to participate

The investigators will ensure that procedures and tasks of the project conform to U.S. and South African ethical standards for human subjects’ research. The study protocol will be reviewed by three independent IRBs (the University of the Witwatersrand, Vanderbilt University and Boston University) to ensure appropriate protocols are set forth for keeping patient data anonymous and confidential. In addition to this, the study protocol will be reviewed by the Bio-specimen and Data Access Advisory Ethics Committee (BDAEC) of the NHLS to ensure the conduct of the proposed study is compliant with the procedures and standards set by the NHLS. A data transfer agreement, setting out conditions of access, will be entered into by the NHLS and the principal investigators (their institutions). Deviations from the set conditions or the protocol will be reported to the NHLS BDAEC as and when they occur.

### Confidentiality

As noted above, we will not collect any data for this study that would not be collected as part of routine care, nor will we have any interaction with patients. All biomedical data for the study will be drawn from routinely collected laboratory databases. We therefore believe that our study poses no physical risks to subjects beyond those routinely encountered. It may, however, pose risks associated with breach of confidentiality and disclosure of HIV status.

#### Disclosure of HIV status and breach of confidentiality

Because no patient interaction will occur in this study, and because no additional data will be collected on or from patients that are not already collected as part of routine care, the main potential risk of the study concerns the confidentiality of data. Data that are used for this study could be lost or stolen. There is always the possibility of a breach of confidentiality when conducting research. While this is acknowledged, there is very low likelihood because of the precautions that will be taken to protect confidentiality. A second potential risk of the study concerns disclosure of data that would reveal someone’s HIV status. A high level of stigmatization continues to inhibit the disclosure of HIV status in the study population. Because the data used as part of this study contain identifying information, the data could be disclosed and reveal a woman’s HIV status. Again, while this risk is real, we consider the risk to be low in light of the precautions we will take to ensure that HIV status of those whose data is used is protected and remains confidential.

#### Protections against risk

Below we describe the steps we will take to protect patients against the risks previously described.

Subjects will be protected against the risk and impacts of accidental disclosure of HIV status and breaches of confidentiality in three main ways. First, we will seek a waiver of informed consent for this study so that no data collected for this study will be able to link subjects to a study of HIV. Second, because these data already exist at the NHLS and no additional data is being generated for the study, the study then poses limited additional risk to patients in the form of accidental disclosure. Finally, all data will be kept strictly confidential. The main data file that links the anonymized data with the full NHLS database will be stored in a password-protected file accessible only to the study team.

To protect against other violations of confidentiality, study staff will be trained in expectations that they are not to disclose any information collected in the study to anyone outside the study team. All study staff will be required to pass an ethics certification course, such as the on-line certification offered by the NIH. As part of that training, we will place additional emphasis on the importance of keeping all data confidential and the required protocol for working only with de-identified data for analysis. All staff outside the NHLS (who own the data) will be educated in the importance of not collecting any identifying information.

Finally, the data shared by the NHLS and the non-laboratory clinical cohorts for validation purposes will not be utilised beyond the aims and objectives as outlined in this protocol. Should any additional analysis or activity outside of the scope of the protocol be deemed necessary, prior approval will be sought from the NHLS and overseeing IRBs.

#### Vulnerable subjects

This analysis will focus on a key vulnerable population - pregnant women. As we will not set specific age ranges, data from adolescents who become pregnant before age 18 will be included in the study. However, their data have already been collected as part of the national program and no contact with subjects will occur. The study team will be aware of the age of each patient whose lab data is included in the dataset as date of birth is included with each lab report in order to identify the patient. However, no other data are collected that would indicate belonging to a stigmatized group. Thus, we believe the risk to these vulnerable populations is minimal.

### Data sharing

The data being used for this analysis are owned by the NHLS, and access is governed by policies and procedures in response to requests made directly to the NHLS Office of Academic Affairs and Research. As such, the study teams at HE^2^RO and VU will not have authority to release the data to the public or other data-sharing repositories. However, these data can be requested by the public through standardized request forms, which are then considered in an internal review procedure. As part of the capacity building component of ongoing work as well as this project, the study team will work closely with NHLS to complete the integration of the record linkage algorithm we developed into the NHLS Corporate Data Warehouse and relevant NHLS subsidiaries. We additionally propose to use the study to support training of a junior data scientist at NHLS who, in addition to other duties, will manage the cohort once internalized at the NHLS, where it can be accessed by researchers complying with the NHLS access procedures. If investigators have specific questions that they wish to ask of the dataset, we are committed to working with outside investigators. We will conduct the analysis for those investigators once the primary questions for this grant application have been answered in accordance with NHLS policies and approvals. In particular, we will work with the South African government to encourage them to ask questions of the dataset, as it has the potential to improve their ability to monitor and improve the national treatment program.

### Dissemination plans

Study findings will be disseminated in several ways. First, we will prepare and submit manuscripts to local and international peer-reviewed journals for publication and present findings at local and international conferences. Second, policy briefs will be made available on the HERO website and shared with the NHLS, SA’s National Department of Health (NDoH), as well as local and international research communities. Third, we will host regular stakeholder meetings with the NHLS and NDoH to disseminate findings from the proposed research. As the sole service provider of laboratory testing services to the public sector in SA, the NHLS represents a significant public health system stakeholder and will be able to utilise the study outputs in direct planning and communication with NDoH. Finally, our plan to incorporate the pregnancy cohort directly into NHLS data systems will make this cohort directly available to scientists and policy makers in SA’s public health system, with real potential to inform clinical practice.

## Discussion

Successful treatment of pregnant women living with HIV has been the cornerstone of reducing vertical transmission of HIV in South Africa. Yet not only does this key group still experience the greatest burden of HIV disease nationally but it is also at the highest risk of becoming lost from care programs at a critical point in their treatment journey. Urgent attention to addressing continuity of care among pregnant women is warranted. The initial steps to improve continuity of care among pregnant women nationally must include more accurate estimations of losses from care than currently available, as well as identification of pregnant women at high risk of loss from care. This will not only reduce losses, but also target efforts to re-engage women in care more precisely and efficiently. We propose to create nothing less than the world’s largest national cohort of women living with HIV, leveraging an innovative data resource already scaled at the national level in South Africa. We will use this cohort to provide the first national estimates of continuity to HIV care both during and after pregnancy and a powerful data tool to identify those women most at risk of disengaging from care. By establishing capacity to incorporate and manage this resource directly within the NHLS, this important cohort can be directly harnessed by the South African National Department of Health to enhance and optimize care of pregnant women living with HIV. In this way, we will ensure the benefits of the initial investment is sustained not only for the duration of the project but also well beyond years of this study.

## Data Availability

The data being used for this analysis are owned by the National Health Laboratory Service (NHLS), and access is governed by policies and procedures in response to requests made directly to the NHLS Office of Academic Affairs and Research. As such, the study teams at HE^2^RO and Vanderbilt University will not have authority to release the data to the public or other data-sharing repositories. However, these data can be requested by the public through standardized request forms, which are then considered in an internal review procedure.

## Abbreviations

AE: Adverse event
AIDS: Acquired immunodeficiency syndrome
ART: Antiretroviral therapy
Hb: Hemoglobin
HE^2^RO: Health Economics and Epidemiology Research Office
HIV: Human immunodeficiency virus
IRB: Institutional review board
NIH: US National Institutes of Health
NHLS: South African National Health Laboratory Service
RPR: Rapid plasma reagin
SAE: Serious adverse event
